# Brainfood cluster

**DOI:** 10.1192/j.eurpsy.2021.170

**Published:** 2021-08-13

**Authors:** R. Adan, S. Dickson

**Affiliations:** 1 Translational Neuroscience, UMC Utrecht, Utrecht, Netherlands; 2 Neuroscience And Physiology, Sahlgrenska Academy, Gothenburg, Sweden

**Keywords:** nutrition, brain health, psychiatry, neurology

## Abstract

**Brainfood cluster** The mission of EBRA brainfood is to increase awareness of the importance of research exploring the bidirectional links between brain health and nutrition, including the mediating systems, and to use this knowledge to identify novel nutritional, neuropsychological and neuropharmacological intervention strategies. The BRAINFOOD cluster builds new bridges across research disciplines and strengthens links to relevant stakeholders across Europe, including those involved in health and food policy. It gathers experts on brain health and nutrition that by combining and integrating strengths and complimentary expertise has the volume and capacity to develop novel intervention strategies that improve brain health of European citizens, working together with public health and the food industry. BRAINFOOD is built upon an existing network that includes: 1 Discovery, with expertise in human genetics, metabolomics, nutrition, the microbiome and brain health that utilizes existing data from a variety of population and disease cohorts across the lifespan and aims to propose testable hypotheses; 2 Mechanism, with expertise in animal models, metabolomics, the microbiome and neuroscience that tests hypothesis of how the microbiome and nutrients impact on performance in different behavioral domains; 3 Experimental medicine, with expertise in psychiatry, neurology and nutrition with capacity to run randomized controlled trials; 4 Implementation, with expertise in dissemination and policy making and behavior change, to ensure that EU citizens benefit from novel insights gained in the project.

**Disclosure:**

No significant relationships.

